# Thermofluidic Nonequilibrium Assembly of Reconfigurable Functional Structures

**DOI:** 10.1021/acsnano.5c05766

**Published:** 2025-06-03

**Authors:** Desmond Joseph Quinn, Diptabrata Paul, Frank Cichos

**Affiliations:** Molecular Nanophotonics Group, Peter Debye Institute for Soft Matter Physics, Leipzig University, 04103 Leipzig, Germany

**Keywords:** thermofluidics, nonequilibrium assembly, self-assembly, functional nanostructures, photonic crystals

## Abstract

Controlled assembly of functional structures that can be dynamically reconfigured remains a significant challenge in materials science. Here, we demonstrate a nonequilibrium assembly approach where colloidal particles organize into three-dimensional crystalline structures through the interplay of three temperature-induced phenomena: thermophoresis, thermoosmosis, and depletion forces from polyethylene glycol molecules. Using precisely controlled laser-induced temperature gradients, we assemble highly ordered colloidal crystals within minutes, significantly faster than conventional equilibrium approaches. These structures exhibit tunable photonic stopbands that can be modulated by adjusting the laser power, causing structural transitions between crystalline and toroidal configurations. By quantifying the underlying particle fluxes and growth dynamics, we develop a model that accurately predicts assembly rates across different conditions. Our thermofluidic assembly approach offers a versatile platform for creating reconfigurable functional materials with dynamically tunable properties, circumventing limitations of traditional equilibrium assembly methods.

## Graphical abstract

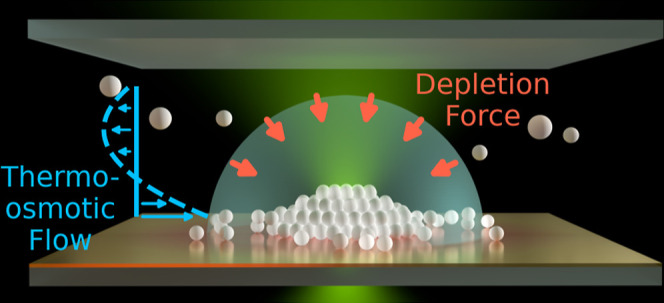

## Introduction

The assembly of simple components into functional structures is a fundamental process across different scales. In biological systems, this is demonstrated through the remarkable hierarchical self-organization of cellular components into complex living structures.^1,2^ In the realm of synthetic materials, simpler yet functional structures can be created through the assembly of engineered units, such as dielectric components that may form, for example, metamaterials.^3^ These synthetic assembly processes are typically driven toward equilibrium states through free energy minimization.^4,6^ Colloidal particle assemblies exemplify this equilibrium-driven approach,^7^ offering various functional capabilities^8^ that enable applications in photonics,^9^ electronics,^10^ chemistry,^11^ and lithography.^12^ Evaporation-driven convection, for example, has been extensively used to induce flows that, in conjunction with capillary forces, can create large-scale photonic crystals.^5,43,55^ Considerable progress has been made in controlling the assembly via particle functionalization, manipulation of the drying mechanism, and tailoring the substrate properties, that finally lead to structure stable even under equilibrium conditions.^53,54^

Alternatively, assembly can also be driven by continuous energy dissipation in nonequilibrium processes, leading to stationary states that are maintained by the energy flux. According to Prigogine’s theorem,^13^ such systems tend toward states of minimum entropy production while maintaining their nonequilibrium character, allowing the dynamic creation and destruction of structures not feasible with equilibrium assembly.^14,15^ These dissipative structures exhibit a well-defined response to external perturbations, leading to new functionalities. In contrast, inhomogeneous temperature fields created by laser heating can drive the assembly of colloidal structures that exhibit random lasing, where the lasing properties can be reconfigured by modulating the laser power.^16^ While techniques enabling local control over assembly through energy flux have remained challenging, they are particularly valuable as they offer the potential to create adaptive structures similar to those found in biological systems. Such approaches could enable reconfigurable structures across diverse scales—ranging from nanoparticles and biological molecules^2^ to living cells and bacteria. Chemical approaches to nonequilibrium assembly, while powerful in their use of dissipative reaction networks,^14,17,18^ are often limited to specific systems and face challenges in achieving precise spatial control. This has motivated the exploration of physical control methods. Particularly, assembly processes driven by external electric and magnetic fields^19–22^ have emerged as an alternative approach, though they too face challenges, primarily in achieving local control over the assembly process rather than applying potentials across an entire region, which only provides bulk control.

Temperature gradients created by localized optical absorption drive persistent flows through continuous energy dissipation,^23–27^ offering a versatile approach to nonequilibrium assembly. This optical control enables highly localized manipulation of matter and, unlike chemically driven systems, can be applied to a broad range of materials. The interplay between different thermally driven processes - such as thermophoresis, thermoosmosis, and convection–creates complex dynamics that can lead to diverse self-organized structures. For example, the competition between optically induced convective flows and thermophoretic drifts has been used to rapidly generate 2D colloidal crystals,^35,52^ though such convective processes require large liquid film thicknesses (several 100 μm) to become effective and rely on inherently static interactions like gravity to form stable structures in the presence of the destabilizing outward convective flows.^48^ Introduction of additional interactions and an understanding and control over the various thermal effects and their interplay is therefore key to developing more versatile assembly strategies that work across different length scales.

Here we report experiments to understand the interplay of thermoosmosis, thermophoresis, and temperature induced depletion of polymers for the dynamic formation and destruction of colloidal structures out-of-equilibrium. We observe rapid colloidal crystal formation under the osmotic pressure generated by the thermophoresis of polyethylene glycol (PEG) molecules and the hydrodynamic forces generated by the thermoosmotic flow. The crystal formation happens despite the thermophoretic repulsion of the colloids from the heat source and has been verified by the measurement of a photonic stop band by angle resolved spectroscopy. At higher temperatures, the thermoosmotic flows break the radial symmetry of the osmotic pressure gradient and lead to the formation of a dynamic toroidal structure that can be collapsed into the crystal structure by a rapid quenching process. Our approach achieves a high degree of control over the assembled structures through time-variable temperature fields induced by lasers in highly localized regions. We explore this structure formation process and report on the emergence of functional photonic properties in the resulting assemblies. These findings provide new insights into controlling nonequilibrium assembly processes and could enable the development of reconfigurable photonic devices.

## Results and Discussion

### Thermofluidic Assembly

The assembly was carried out in an inverted microscope setup that uses an Acousto-Optic Deflector (AOD) to control and modulate a 532 nm wavelength laser spot that was focused to the sample plane by a oil-immersion objective lens (100×, NA 0.5–1.35). The focused laser spot enables local heating of a gold film (50 nm) by optical absorption. The resulting temperature was calibrated using a liquid crystal method^24^ and substantiated using finite-element simulations (detailed in the Supporting Information). The sample was imaged in real space using dark-field illumination and in Fourier space via a Bertrand lens^28^ in two separate imaging paths. The Fourier space image which corresponds to the Back Focal Plane (BFP) of the objective lens contains the angle resolved information for a wavelength range selected by a variable band-pass filter placed along the path. Further, an adjustable slit and flippable grating added to the Fourier imaging path was used to obtain spectroscopic information by dispersing the light before being captured by the camera (see Figure 1A). The assembly was carried out for polystyrene (PS) particles [345, 400, and 442 nm diameter; measured via Dynamic Light Scattering (DLS)] in a solution of 7% (w/v) Polyethylene glycol 6000 (PEG). The colloidal solution was placed between two glass coverslips maintained at 5 μm distance to facilitate growth in 3 dimensions (see Figure 1B).

**Figure 1:**
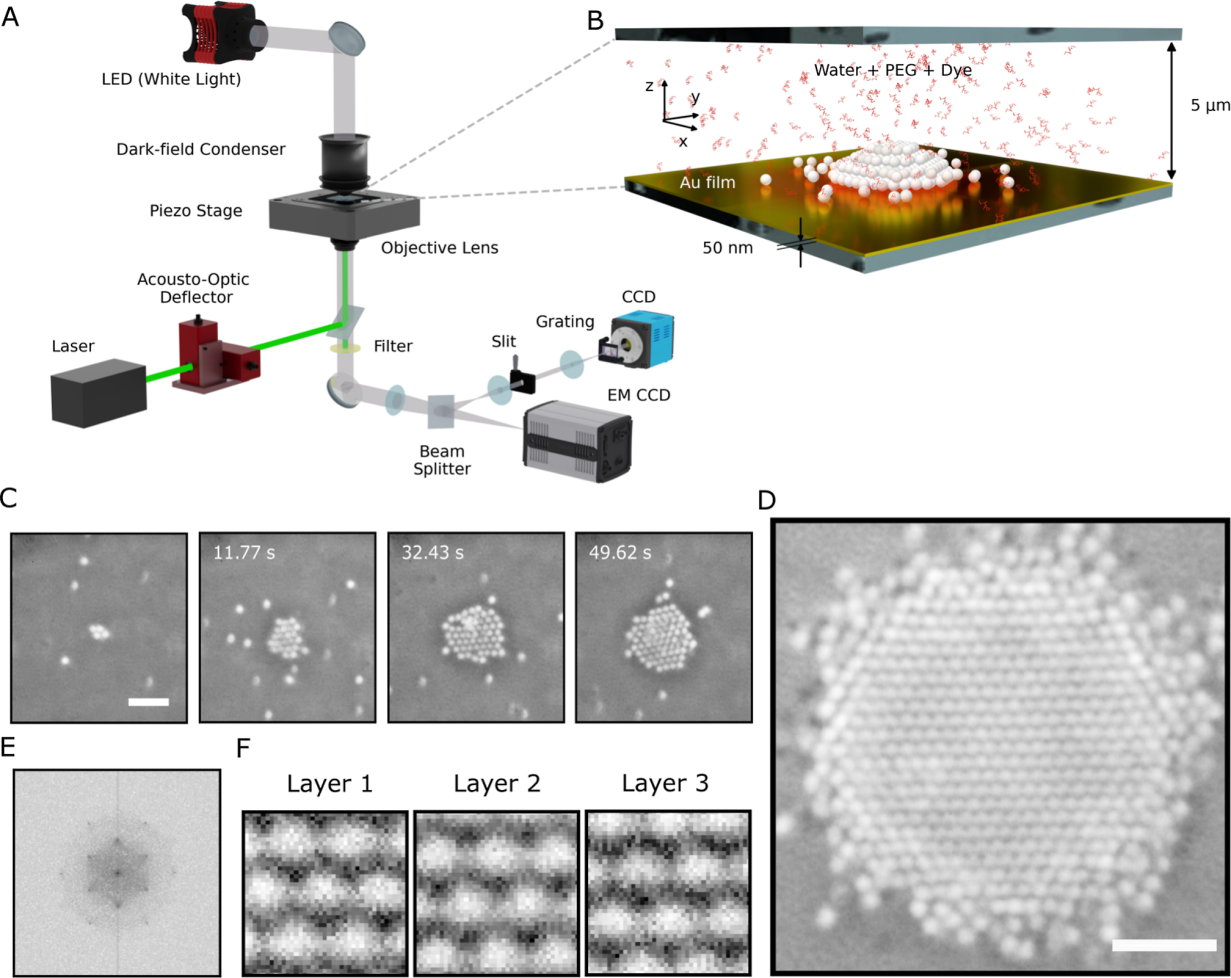
Thermofluidic assembly of a 3D colloidal crystal. Polystyrene (PS) particles with diameter 442 nm are assembled in polyethylene glycol (PEG) solution by laser-induced temperature gradients (A) The experimental setup comprises of a steerable laser of wavelength λ = 532 nm that is controlled by an acousto-optical deflector in an inverted microscope that images the sample in both real and Fourier space. The Fourier imaging path contains an additional flippable slit and grating for measuring spectra. (B) The sample comprises a 5 μm thick liquid film (water + PEG) between two glass coverslips, of which the bottom coverslip is coated with a 50 nm gold film. The gold film enables local heating in the sample via optical absorption of the focused laser of wavelength λ = 532 nm (C) Series of darkfield images of the assembly of a colloidal crystal at *P*_0_ = 0.26 mW. (D) Darkfield image of the highly ordered crystal. (E) Fourier transform of (D) displaying the 6-fold symmetry. (F) The different layers of the colloidal crystal, imaged by focusing through the structure with a piezoelectric stage is shown. (Scalebar in (C,D) is 3 μm).

### Experimental Growth Dynamics of the Colloidal Assemblies

In the absence of any heating of the gold film, the 442 nm PS particles carry out Brownian motion with a diffusion coefficient of *D* = 0.38 μm^2^ s^–1^ that has been evaluated experimentally by single particle tracking. The observed value is approximately one-third of the bulk value, a reduction attributable to well-documented hydrodynamic effects that occur in proximity to a surface. Increasing the laser power to *P*_0_ = 0.26 mW leads to a local heating of the gold film and a steady radial influx of particles yielding the assembly of a crystalline 2-dimensional­(2-d) colloidal structure at the gold surface as displayed examplarily in Figure 1C for 420 nm PS particles. When a larger 2d structure has formed, particles also start to occupy the interstitial sites on top of the 2-d layer to form a 3-dimensional (3-d) crystal. A highly crystalline order was observed (see Figure 1D,E). The crystal structure is well identified in the optical images for 442 nm particles and reveals an FCC stacking as measured by focusing through the crystal structure using a piezo-electric stage (see Figure 1F). The measured distance between successive layers in the assembled structures also corresponds to *d*_111_, the spacing between the (111) lattice planes of such an FCC crystal. When the heating laser is turned off, the crystal structure disintegrated, causing the particles to disperse through Brownian motion.

The crystal growth kinetics varies with heating power and can be quantified by tracking the structure’s area expansion over time. We selected area measurements as our primary metric because they can be directly extracted from microscopy images, whereas volume determination is challenging due to the optical point spread function exceeding the dimensions of our smallest colloidal particles. Figure 2A illustrates the growth dynamics of the assembled structure for 345 nm PS particles at an incident laser power of *P*_0_ = 0.26 mW, which corresponds to a maximum temperature increment of Δ*T*_max_ = 6 K directly at the gold film (see Video S1 in the additional information). The area *A* was found to grow as a power law given by

1 $$A\left(t\right)={\left[k\left(t+t_{0}\right)\right]}^{\alpha }$$

having exponent $\alpha =\frac{2}{3}$ (Figure 2A). A growth rate *k* = 11.16 μm^3^ s^–1^ was obtained for the corresponding growth by fitting the measured area to the aforementioned power law. After 82.5 s, a crystal structure with an area of *A* = 106.1 μm^2^ and several layers was formed.

**Figure 2:**
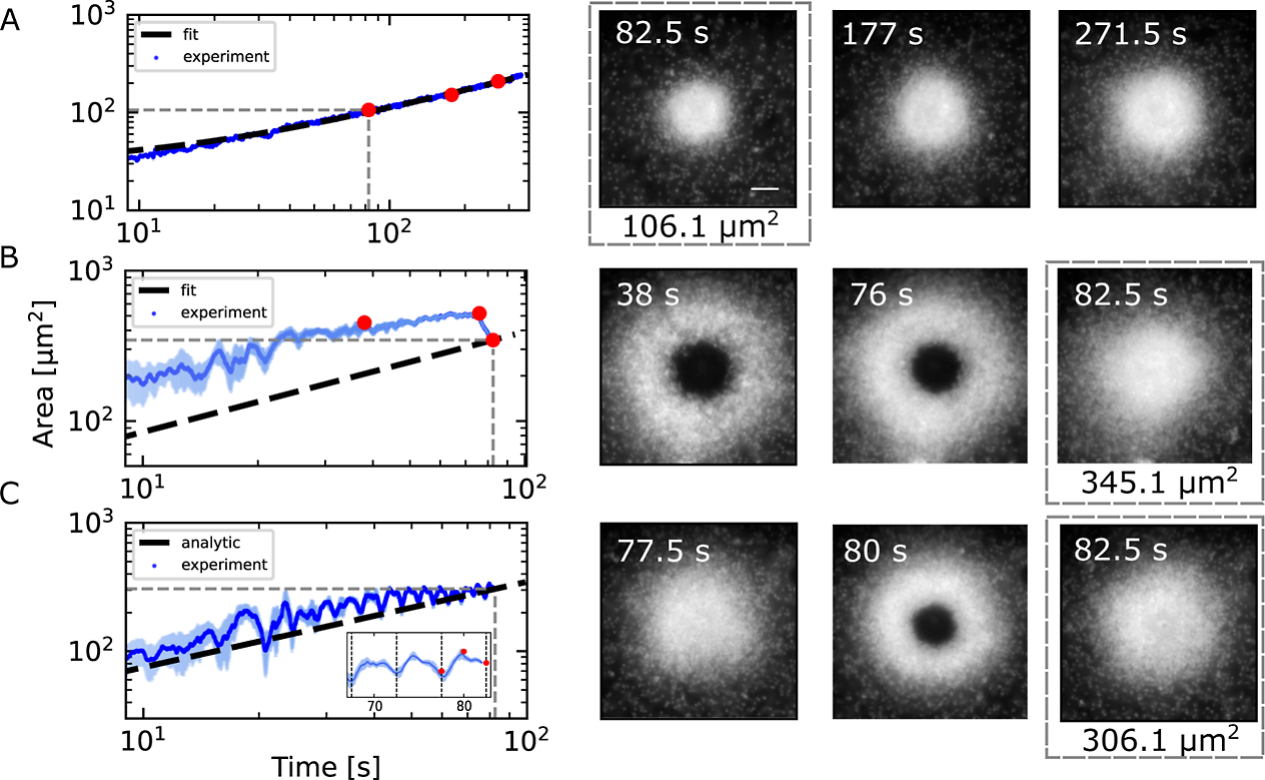
Colloidal assembly dynamics under different laser heating modes. Assembly of 345 nm polystyrene (PS) particles using three distinct laser heating protocols controlled via an acousto-optical deflector. The temporal evolution of the assembled structure’s area directly above the gold film was tracked, with representative microscopy images shown at key time points (marked in red). (A) Assembly under constant laser power (*P*_0_ = 0.26 mW). The growth curve is fitted by eq 1 with the parameters $\alpha =\frac{2}{3}$ and the growth rate *k* = 11.16 μm^3^ s^–1^. (B) Two-stage assembly: initial structure formation at elevated power (*P*_1_ = 2.16 mW) for 76 s, followed by crystallization at reduced power (*P*_0_). The dashed line indicates the effective growth curve that yields the final measured area according to eq 1, and produces an effective growth rate *k* = 77.7 μm^3^ s^–1^ that allows us to quantify and compare the assembly rates even at higher powers and other growth modes. (C) Assembly under pulsed laser heating, alternating between *P*_1_ and *P*_0_ with a 10 s cycle time. The dashed line indicates the effective growth curve that yields the final measured area according to eq 1, and yields an effective growth rate *k* = 64.91 μm^3^ s^–1^. The inset reveals periodic area oscillations matching the laser pulse frequency, with corresponding structural transitions shown in the images. All growth curves represent 50-frame moving averages of experimental data. (Scalebar for all images is 2 μm).

A further rise in laser power and temperature results in a stronger radial influx of particles and accelerated growth. Above a certain temperature increment of Δ*T*_c_ = 10 K, however, there are qualitative differences, as the resulting ensemble structure is no longer a solid crystal, but highly dynamic (see Video S2 in the additional information). A circular zone opens up in the center where the particles get depleted. The radius of which further increases with increasing heating power.

Figure 2B displays the growth of an assembled structure of 345 nm PS particles at a constant laser power of *P*_1_ = 2.1 mW (Δ*T*_max_ = 48.8 K). The image at *t* = 38 s depicts the hole of 5.6 μm radius at the center of the assembly, while at *t* = 76 s, the assembly appears significantly denser with a smaller hole (4.4 μm radius) and a larger surface area. When we reduce the laser power back to *P*_0_ = 0.26 mW, the structure rapidly collapses into a dense ensemble within seconds (Figure 2B). This rapid quenching process results in a final area of 345.1 μm^2^ at *t* = 82.5 s – 3.25 times larger than the area achieved when assembling with constant *P*_0_ power for the same duration. From the final structural area, we extract an effective growth rate of *k* = 77.7 μm^3^ s^–1^ according to eq 1. This rate, shown as a dashed line in the figure, characterizes the kinetics necessary to produce the observed final structure.

The rapid quenching was repeated in a cyclic manner to switch between a highly dynamic toroidal structure and a dense solid structure by switching between the heating powers *P*_0_ and *P*_1_ as indicated in Figure 2C for a cycle time of 10 s. The cycle time here refers to the duration of a complete power oscillation. Consequentially, the higher power *P*_1_ is activated for a duration of 5 s and subsequently decreased to power *P*_0_ for 5 s before the higher power *P*_1_ is reactivated and the cycle continues. During cycles of low and high heating power, the structure is still growing (see Video S3 in the additional information).

After 82.5 s, the collapsed state covers an area of 306.1 μm^2^, which is 2.9 times higher than the steady growth case, and corresponds to an effective growth rate of *k* = 64.91 μm^3^ s^–1^. At larger times, the corresponding growth curve contains periodic oscillations with frequency corresponding to half the cycle time of the laser power (see inset of Figure 2C). The appearance of prominent peaks suggest that once a large number of particles have accumulated, a dense, 3-dimensional structure can be formed and destroyed at the center in a matter of seconds.

### Emergence of Photonic Property

Distinct properties can emerge when colloidal nanoparticles are assembled into ordered structures. Dielectric structures exhibiting spatial periodicity modulate the propagation of electromagnetic waves due to the photonic band structure dictated by their crystal structure. In particular, stop bands, i.e. directions in which light propagation is forbidden for certain wavelengths, can emerge.

Determining the stopbands of colloidal crystals traditionally requires angle-resolved light transmission measurements, typically obtained by physically tilting the sample.^44^ This approach presents challenges in microscopy configurations and risks disrupting delicate reconfigurable assemblies. To address these limitations, we implemented a noninvasive single-shot method by imaging the back focal plane (BFP) of the objective lens, which inherently provides angle-resolved information on the transmitted light.^29^ Dye molecules embedded inside the photonic crystal with a suitable absorption/emission characteristics provided the necessary emission inside the crystal (see Materials and Methods). The fluorescent light emitted by these internal emitters propagates through the assembled crystal structure and is modified by its photonic properties (Figure 3A).

**Figure 3:**
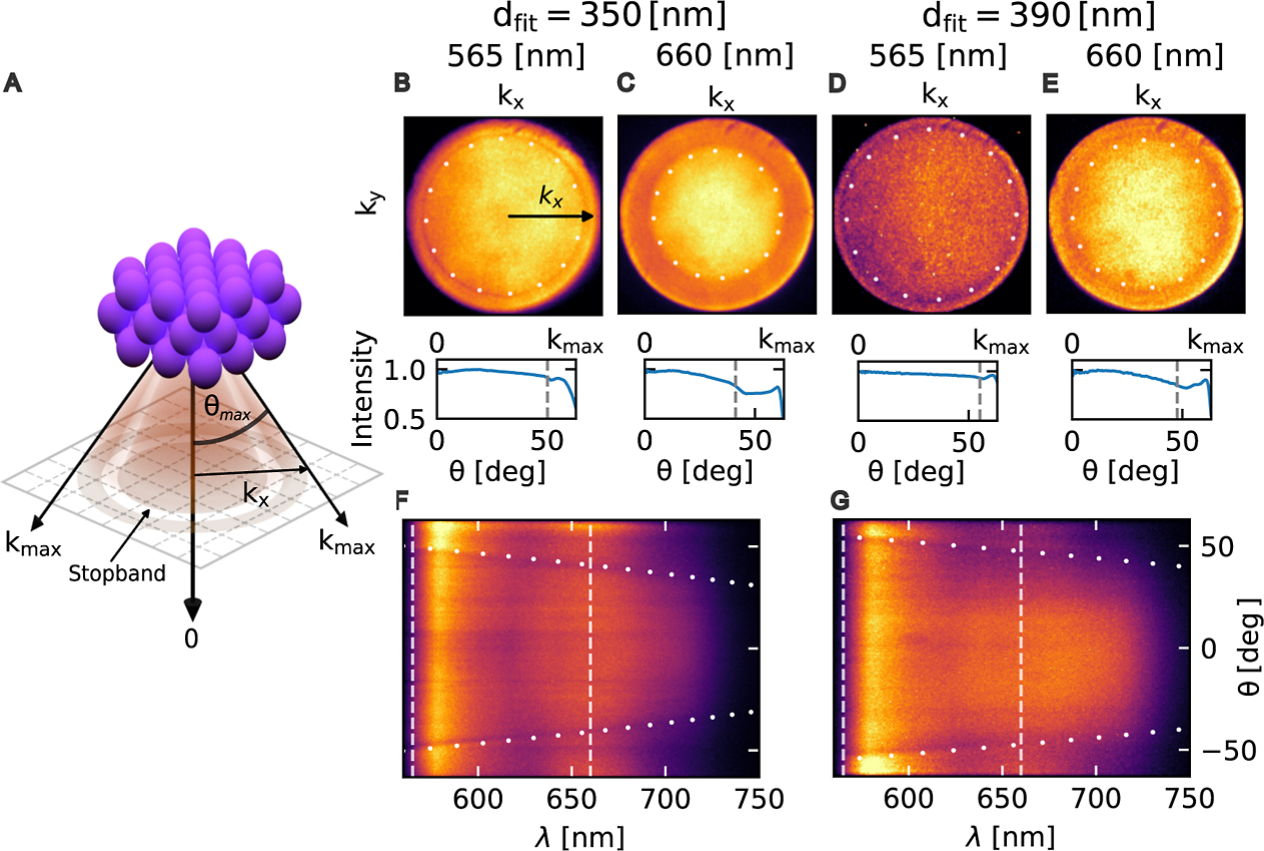
Characterization of photonic stopbands in colloidal crystals. Analysis of photonic stopbands in assembled crystalline structures using Fourier plane imaging and spectroscopy of embedded fluorescent emitters. (A) Schematic showing light propagation through the colloidal crystal from embedded emitters. (B–E) Fourier plane images and corresponding radial intensity profiles for assembled polystyrene (PS) spheres of different diameters: (B,C) 345 nm and (D,E) 400 nm. Dark bands in the Fourier images, obtained using different band-pass filters (wavelengths indicated), correspond to photonic stopbands. White dotted curves show theoretical fits using the Bragg equation, yielding fitted particle diameters (*d*_fit_) that closely match nominal sizes. (F,G) Back focal plane (BFP) spectra for crystals assembled from (F) 345 nm and (G) 400 nm PS particles, with dashed vertical lines indicating wavelengths corresponding to the Fourier images shown above. The spectral measurements confirm the wavelength-dependent behavior of the photonic stopbands.

The BFP images of assembled crystals containing embedded dye molecules contained evidence of altered photonic properties. The BFP images (Figure 3B–E), exhibited dark circular bands corresponding to certain wavevectors (angles), implying the presence of photonic stopbands in the assembled structures. The radial intensity profiles further highlight this dip in intensity. Using different optical filters shifted the position of the dark bands, moving to smaller wavevectors (angles) for larger wavelengths. The measured BFP spectra (Figure 3F,G) revealed the trend of the stopband with varied angles and wavelengths. Further, the differently size particles displayed different stopband positions.

The wavevectors imaged in the BFP correspond to light incident at different angles on the plane parallel to the substrate, which corresponds to the (111) plane for the assembled FCC crystal structure. The interference of the scattered light from the set of parallel (111) crystallographic planes give rise to Bragg peaks, analogous to the Bragg peaks seen in X-ray diffraction experiments on atomic crystal lattices. The Bragg peaks correspond to high reflectivity, and result in lower intensity of collected light. The Bragg condition for such an optical system is given as^30^

2 $$2d_{hkl}n_{0}\cos\left(\theta \right)=m\lambda$$

where *d*_*hkl*_ is the interplanar spacing, *n*_0_ is the effective refractive index, θ is the angle of emission, and λ is the wavelength. We use *d*_*hkl*_ = *d*_111_ = 0.816*D*, which corresponds to the spacing of the (111) planes of the FCC crystal, with *D* being the diameter of the particles used. The effective refractive index *n*_0_ was approximated as *n*_0_ = *f*_p_ *n*_p_ + *f*_m_ *n*_m_,^31^ where *f*_p_ and *f*_m_ are the filling fractions of the particles and medium, respectively, and *n*_p_ and *n*_m_ are their corresponding refractive indices. The size of the colloidal particles sets the interlattice spacing which in turn determines the stopband position and resulting spectra as seen in eq 2. Hence the stopband position could be tuned via the particle sizes. The contrast (or strength) of the photonic stopband is determined by the refractive index contrast between the colloidal particles and the surrounding medium. Specifically, in these periodic dielectric structures, both the width and depth of the stopband increase with greater refractive index contrast. For the relatively modest index contrasts in our colloidal crystals, this relationship is approximately linear with the relative index contrast (*n*_p_/*n*_m_ – 1), with the exact scaling depending on the crystal structure, filling fraction, and number of unit cells in the beam path. The stopbands obtained in our experiments were found to be in good agreement with the predictions of the Bragg equation (plotted in Figure 3 as dotted lines). Further, the trend of the wavelength dependence of the stopband obtained from the BFP spectra also matched well with the Bragg predictions. The presence of the stop band is also an indicator of crystallinity, that is otherwise hard to resolve for the smaller 390 and 400 nm PS particles.

We measure the stopband when the laser was cycled between a high and low heating power *P*_0_ and *P*_1_ with a cycle time of 8 s (refer Figure 4A for microscopy image, B for stop band). Since the photonic stopband emerges from the crystallinity of the structure probed by the fluorescence at the focus of the laser, it disappears when the crystal structure is destroyed at the center by high temperatures. The oscillating crystallinity at the center resulted in an oscillating photonic stopband Figure 4. The photonic stopbands disappeared whenever the crystal was destroyed in the center by the formation of the dynamic toroidal structure, but reappeared whenever the colloids were collapsed back into a crystal structure. The presence of the stopband in the collapsed structures elucidates that the crystallinity is still preserved in structures formed by the rapid quenching process. In spite of the rapid nature of the quenching process, the diffusion of the nanoparticles during the time-scale of the collapse is still high enough to allow for structural relaxation required to prevent the freezing of defects typically associated with quenching processes. We found that employing these accelerated growth dynamics (Figure 2B,C) does not adversely impact the crystallinity of the final structures, enhancing speed without compromising quality.

**Figure 4:**
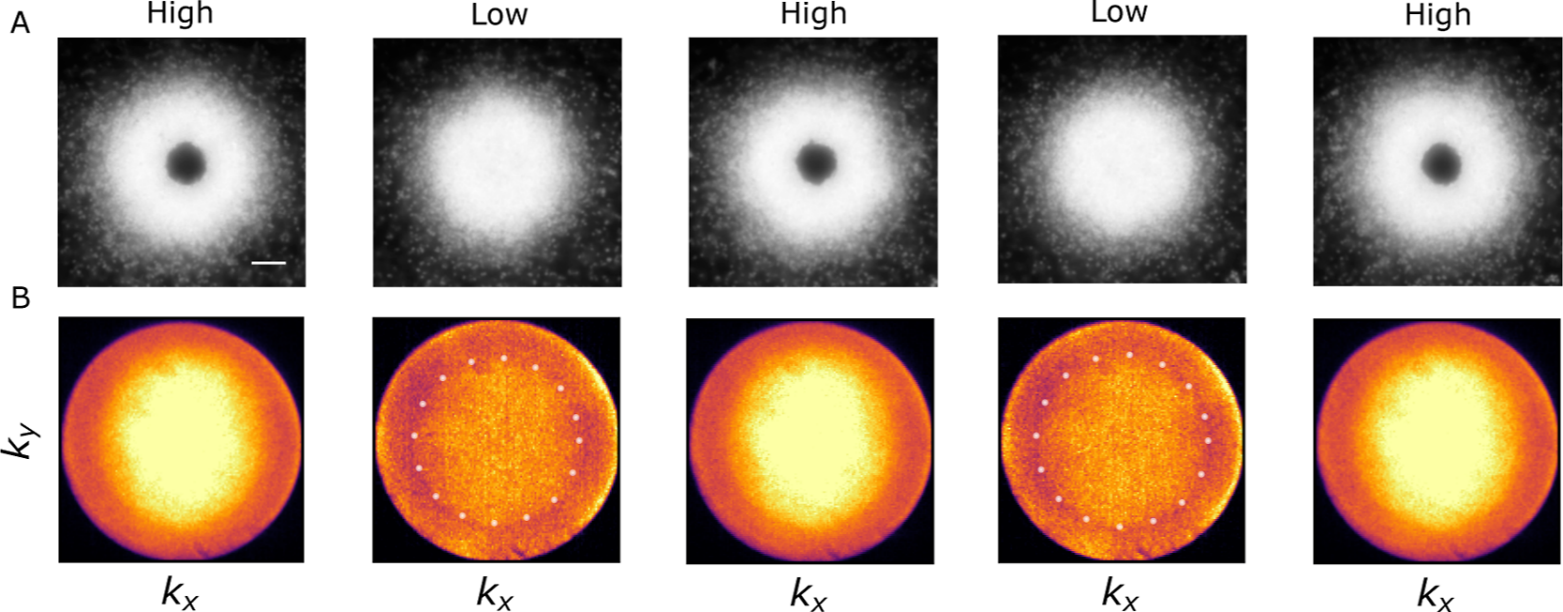
Dynamic modulation of photonic stopbands through pulsed laser heating. Analysis of photonic stopband variations in colloidal structures assembled from 345 nm polystyrene (PS) particles under oscillating laser power (*P*_1_ = 2.16 mW to *P*_0_ = 0.26 mW, 8 s cycle period). (A) Real-space microscopy images showing structural transitions at the midpoints of high and low laser power phases. (B) Corresponding Fourier plane images (measured using a 660 nm band-pass filter) revealing stopband modulation between power phases. White dotted curves represent Bragg equation fits, yielding an effective particle diameter *d*_fit_ = 350 nm.

### Discussion

When the tightly focused laser spot (beam waist 250 nm) is absorbed by the gold layer, it generates heat locally. This results in a stationary inhomogeneous temperature profile in water that is reached within a time duration of a few tens of microseconds^32^ due to the high thermal diffusivity of water (α_water_ = 0.14 × 10^–6^ m^2^ s^–1^). The resulting temperature gradients can drive a flow of particles toward the heated area due to various thermofluidic effects.

The temperature gradients at the substrate–solvent interface perturb the liquid–solid interactions and result, among other effects, in thermoosmotic flows.^24^ The flow field inside the liquid film can then be calculated from the hydrodynamic boundary condition at the gold liquid interface with the slip velocity

3 $$u_{TO,slip}=-\frac{1}{\eta }\int_{0}^{\infty }zh\left(z\right)\;dz\;\frac{\nabla T}{T}=\chi_{AuL}\frac{\nabla T}{T}$$

where η is the viscosity of the liquid, *h*(*z*) is the excess free enthalpy density, *T* is the temperature, ∇*T* is the temperature gradient at the interface. χ_AuL_ is the thermo-osmotic coefficient at the Gold-liquid interface, which takes a value of 10 × 10^–10^ m^2^ s^–1^ as previously measured for the gold water interface.^24^

The temperature gradients across the solute particles and molecules in the liquid also cause a thermophoretic drift current density.^33,34^ The corresponding drift velocity **v**_TP_ of the objects undergoing thermophoresis is described by the equation

4 $$v_{TP}=-\frac{2}{3}\chi_{SL}\frac{\nabla T}{T}=-D_{T,i}\nabla T$$

where χ_SL_ is the osmotic coefficient at the solid–liquid interface, which gives rise to the thermophoretic mobility of the species *D*_T,i_, where *i* = (*C* = PS, *P* = PEG), which take on values *D*_T,C_ = 0.3 μm^2^ K^–1^ s^–1^ ^24^ and *D*_T,P_ = 5.54 μm^2^ K^–1^ s^–1^.^36^ The thermophoresis alters the distribution of the PEG molecules, which reaches a steady state when the thermophoretic and diffusive molecule fluxes balance, resulting in a concentration profile described by eq 5.

5 $$c_{P}\left(r\right)=c_{0,P}\exp\left(-S_{T,P}\Delta T\left(r\right)\right)$$

where *c*_0,P_ is the initial concentration of PEG, *S*_T,P_ = *D*_T,P_/*D*_P_ = 0.05 K^–1^ is the Soret coefficient of the PEG molecules.^36^ The thermally generated concentration profile results in an excess osmotic pressure that acts on the PS particles. For an interaction potential ϕ between the PS particles and solute molecules the osmotic pressure can be calculated by

6 $$\Pi \left(r\right)=c_{P}\left(r\right)k_{B}T\left(e^{-\phi /k_{B}T}-1\right)$$

where *k*_B_ is the Boltzmann constant, and yields a drift of the particles toward the heated spot.^25,26,37^ The drift velocity of the particles due to this depletion interaction is then given by^38^

7 $$v_{D}\left(r\right)=\frac{k_{B}}{3\eta }R_{P}^{2}c_{P}\left(r\right)\left(TS_{T,P}-1\right)\nabla T$$

where *R*_P_ = 3.62 nm is the radius of gyration of the PEG molecules used.

Although temperature gradients can induce natural convection that drives particle assembly,^48–50,52^ this mechanism is negligible in our system. The small height of our sample (5 μm) yields a Rayleigh number of approximately 10^–5^, several orders of magnitude below the critical threshold (typically ∼10^3^) at which buoyancy forces overcome viscous resistance characterized by the Rayleigh number. The assembly of the colloidal particles as observed in the experiment depends on the interplay among the thermofluidic effects. According to eqs 3, 4 and 7 all of these drifts depend on the gradient of the temperature field, yet, eq 3 only refers to the temperature gradient along the solid–liquid boundary. The thermophoretic and the diffusiophoretic drifts (eqs 4 and 7) follow the symmetry of the temperature field. The velocities are perpendicular to the temperature isotherms and can be either along the temperature gradient (attractive to the heat source) in the case of diffusiophoresis induced drift (eq 7) or against the temperature gradient (repulsive with positive *D*_T_) in the case of thermophoresis (eq 4). Thus, any change in the heating power and the maximum temperature would only yield a quantitative change in the distribution of molecules and particles in the system, but no qualitative change as observed in the experiment when changing from low to high powers.

The thermoosmotic boundary flow described by eq 3, however, resembles a boundary slip flow that drives the flow field in the liquid film. The drag forces acting on the particles and molecules therefore do not follow the temperature gradient direction everywhere in the liquid. In particular, the flow field exhibits an upward velocity in the *z*-direction above the hot spot and a radial outflow in about the middle of the liquid film due to mass conservation. As it is driven by osmotic pressure differences, the flow follows a parabolic radial profile as reported previously.^23,24^ The thermoosmotic flow therefore breaks the symmetry of the temperature field and will give rise to new qualitative behavior if the flow generates drift velocities on the particles that are of the same order of magnitude or higher than the other drift velocities.

This qualitative change can be substantiated by a numerical calculation of the total thermofluidic drift velocities (eqs 3, 4, and 7) for the two different experimental optical heating powers. Figure 5A,B show the calculated drift field for the two used powers *P*_0_ and *P*_1_. While for *P*_0_ all drift velocities point radially inward to the heat source, Figure 5B reveals a reversal of the sign of the radial drift speed *v*_r_ for *P*_1_. Figure 5C,D decomposes the contributions showing the normal component *v*_z_(*z*) of the drift velocities for a point-like particle located at *x* = 0. When Δ*T*_max_ = 6 K for *P*_0_, the total velocity vectors are always directed toward the heated spot. As visible in Figure 5C the attraction to the hot spot is caused by the PEG gradient while thermo-osmotic flow and thermophoresis play only a minor role. Switching to *P*_1_, i.e to Δ*T*_max_ = 48.8 K causes a stronger contribution of the PEG gradient to *v*_z_ but also stronger thermo-osmotic flows. The total speed *v*_z_ therefore points away from the heated spot at the center (seen as positive velocities) up to a distance of *z* = 2.2 μm, beyond which it continues pointing in the direction of the heated spot. Yet, the zero drift speed in the vertical direction is not a stable point in the flow field due to the thermo-osmotic outflow in the middle of the film. Due to this outflow, a toroid-like structure appears in which the particles are confined (yet dynamic) and which is clearly visible in the experiment (see Movies S2 and S3). The particles follow the outflow, leaving a void in the center, but loop back due to flow profile and the depletion contribution that becomes more relevant further from the center where the osmotic outflow is weaker or not present. Figure 5E highlights this transition from an attractive to a repulsive flow in the vertical *z*-direction again for different maximum temperature increments Δ*T*_max_. Plotting *v*_z_ at *z* = 0.16 μm (Figure 5F) indicates that initially the attraction is enhanced and then reverses to a repulsive behavior (*v*_z_ > 0 μm s^–1^) at Δ*T*_max_ = 32.5 K for this position. Yet, at *z* = 4.5 μm we still find a linear increase of the attractive speed *v*_z_ < 0 μm s^–1^ toward the heated region from the numerical calculation (Figure 5G). The heated region therefore continues to attract colloidal particles, but the colloids do not reach the central hot spot, instead becoming trapped in the toroid structure as found in the experiment as well.

**Figure 5:**
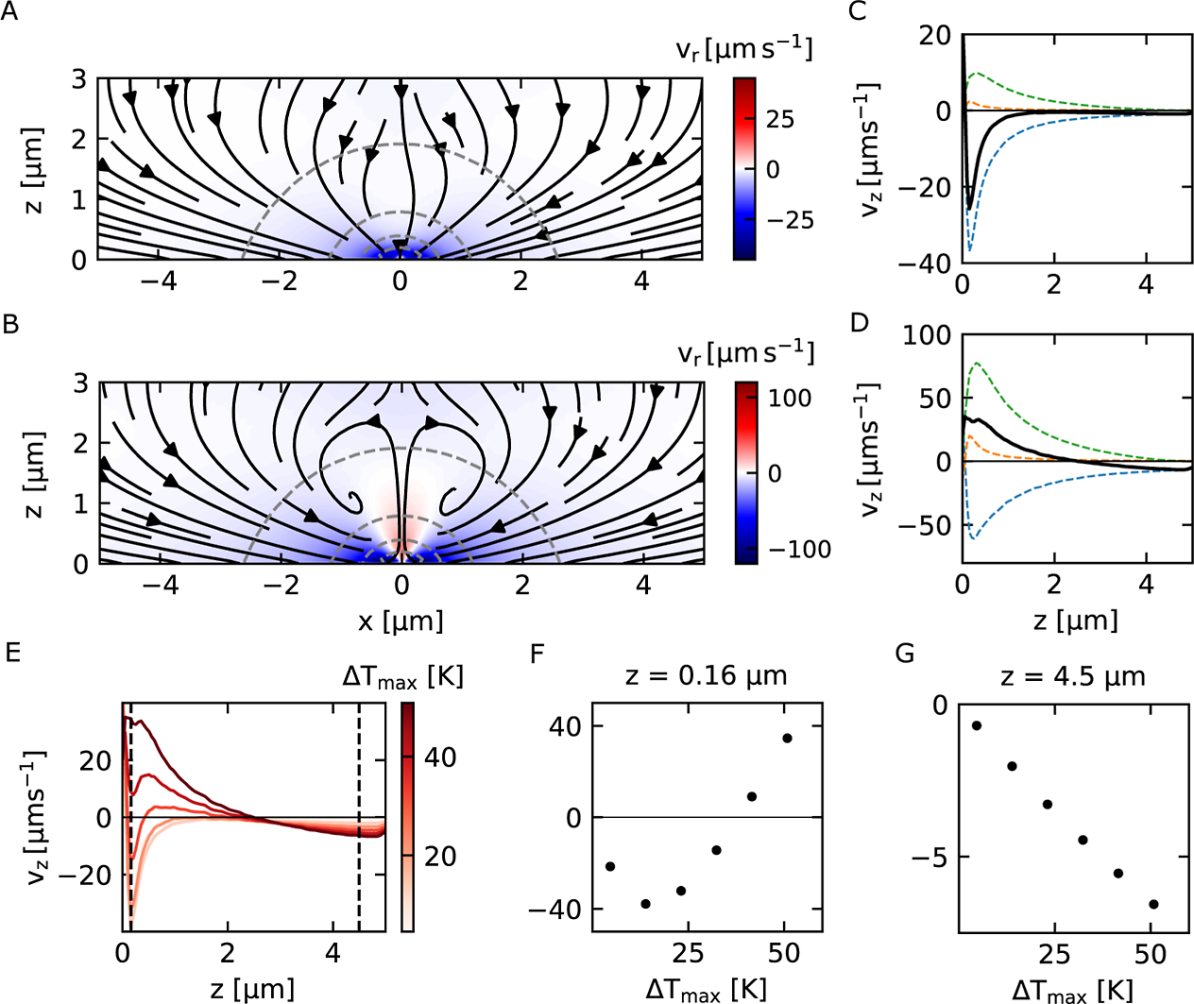
Thermofluidic velocity fields under varying laser powers. Numerical simulation of velocity fields for a point-like particle arising from combined thermofluidic effects (thermoosmosis, thermophoresis, and depletion) under different laser heating conditions. (A) Radially symmetric inward velocity field at low laser power (*P*_0_ = 0.26 mW). (B) Asymmetric velocity field at high laser power (*P*_1_ = 2.16 mW), showing emergence of a central repulsive region. Color maps in (A,B) represent radial velocity magnitudes. (C,D) Vertical components of individual contributions (thermophoresis: red, thermoosmosis: blue, depletion: green) and net velocity (black) along x = 0 for powers *P*_0_ and *P*_1_, respectively. (E) Net velocity fields mapped across different temperature increments. (F) Temperature-dependent velocity profiles at z = 0.16 μm, showing flow reversal (away from heat source) at high temperatures. (G) Velocity profiles at z = 4.5 μm, demonstrating consistently inward-directed flow with monotonically increasing magnitude at higher temperatures.

The flow speeds obtained from the numerical calculations of the different contributions can now be used to quantify the crystal growth. For this purpose we consider the growth at low power where all particles drifts are toward the heart source (Figure 5A). For the measured area above the interface, the inflow of particles due to the drift tangential to the interface is important. The phenomenological equation for this particle inflow can be written as

8 $$j_{c,t}=v_{c,t}c_{c}$$

where **v**_c,t_ corresponds to the tangential drift velocities of the colloids that can be found by averaging the drift velocities from the underlying thermofluidic processes (eqs 3, 4, and 7) obtained from numerical calculations over the distance in z explored by the colloids between successive camera frames Δ*z* = 2*R*_c_ + *D*_z_ = 0.45 μm, where *R*_c_ = 0.17 μm is the radius of the colloidal particle and *D*_z_ = 0.1 μm is the diffusion length in *z* of the colloids at the interface in this time interval.

Based on the constant influx of particles to the central region at low heating power, the conservation of particle number dictates the growth of the observed crystal by a power law (eq 1) with an exponent of α = 2/3. The value of the exponent is characteristic of the temperature gradient profile of the point-like heat source driving the assembly. The rate constant quantifying the crystal growth is related to the different drift velocities as

9 $$k=\frac{4\pi^{5/2}R_{c}^{3}v_{c,t}^{0}c_{c}}{\eta_{pack}}$$

where *v*_c,t_^0^ is the magnitude of the tangential velocity at the edge of the heat source (taken as *x* = 0.3 μm), *c*_c_ = 0.8 μm^–3^ is the number density, and η = 0.74 is the packing fraction of the colloidal particles. The above equation illustrates that changing the magnitude of the velocities (via the laser power) increases the growth rate constant as it scales the gradients. The exponent which is dictated by the profile of the temperature gradients rather than the magnitude is not altered when the laser power is changed. The derived power law is similar to the Ostwald coarsening process^39^ and was also observed in experiments where passive particles were assembled by phoretic fluxes induced by active particles.^40^ For the lower temperature increments Δ*T*_max_ = 6 K, we calculate *v*_c,t_^max^ = 25.0 μm s^–1^, resulting in a growth constant *k* = 9.6 μm^3^ s^–1^, in close agreement with the value fitted from the experimental curve (see Figure 2A).

For a temperature increment of Δ*T*_max_ = 48.8 K, which is 8.13 times higher, the growth constant *k* would increase proportionally since velocities outside the central region scale linearly (see Figure 5G). This would yield an area 8.13^2/3^ = 4.04 times larger than the previous case at the same time point. In our experiments, the measured growth rate *k*, calculated from the final area, was 3.25 times higher–slightly lower than the model’s prediction. This discrepancy could be explained by the reduced velocities in the experiment near the center at higher laser powers, which would result in the area prediction of the model being higher. Despite this small difference, our model successfully captures how stronger hydrodynamic fluxes affect structure size at higher temperature increments and provides a framework for quantifying particle accumulation. The model unifies various thermofluidic processes through the velocity parameter *v*_c,t_^0^, which emerges as the key factor controlling growth in a given sample and is experimentally accessible via the laser power.

With the quantitative understanding of the interplay of temperature-related forces and flows at a local level—including thermophoresis, thermo-osmosis, and temperature-based depletion—we can guide individual components to come together and form structures. This allows us to accurately control the assembly dynamics and kinetics by varying the input energy or the assembly components. Changing the input energy via the laser power not only changes the kinetics of the assembly but also the nature of the structures, with new dynamic structures, unique to nonequilibrium assembly, emerging when we break their symmetry via the temperature.

The ability to precisely control local assembly through temperature gradients opens new possibilities for creating adaptive materials with programmable properties. The interplay between different thermofluidic effects demonstrated here suggests that even more complex assembly modes could emerge when working with mixtures of particles of different sizes, shapes, or surface properties. For instance, particles with different thermophoretic responses could self-sort into distinct regions, while asymmetric particles might orient themselves along the temperature gradients, potentially leading to hierarchical structures. The dynamic nature of the heating could be exploited to create temporal patterns in the assembly process, enabling sequential organization of different components or the formation of gradient structures.

While the dynamic structures reported here arise due to thermofluidic interactions of simple spherical colloids, many more unique dynamic structures could arise from additional interactions in more complex colloids or mixtures.^47^ For example, particles with different surface chemistries might exhibit competing attractions and repulsions, leading to the formation of novel spatiotemporal patterns. Our approach differs significantly from traditional colloidal crystal formation methods that rely on evaporation-driven convection and capillary forces,^43^ which, while effective for producing large-scale colloidal crystals, lack the dynamic control and reconfigurability achieved through our thermofluidic assembly. Unlike evaporation-based methods that progress toward irreversible dried states, our temperature-gradient driven assembly maintains the system in a fluidic environment where structures can be dynamically modulated.

The potential of this approach could be further enhanced by combining it with other advanced assembly techniques. By harnessing the precise programmability of DNA origami, hierarchical assembly of complex 3D lattices^45^ and scaffolds for diamond lattices that possess complete bandgaps could be obtained.^46^ The speed of heat diffusion being orders of magnitude higher than particle diffusion^32^ would allow for parallel assembly of structures,^24^ which would make such colloidal assembly even faster.

Furthermore, this assembly method could be extended beyond colloidal systems. The material library could be extended to include other soft materials, and even molecules like block copolymers for the development of novel, reconfigurable photonic structures.^51^ The use of higher refractive index colloids or lower refractive index medium could improve the stopband contrast for better performance in optical applications, while the physical mechanisms of assembly remain valid, though additional optical forces may play a role. Beyond colloidal particles, new modes of accumulation could be implemented for molecules or analytes of interest, potentially enabling applications in chemical sensing or molecular separation. In biological contexts, components such as cells that are inherently active could organize into even more complex and dynamic patterns, suggesting possible applications in tissue engineering or research into cellular organization. Precise spatial and temporal control over temperature gradients could be particularly valuable for coordinating the assembly of temperature-sensitive biological components or creating biomimetic structures that respond to environmental cues.

## Conclusion

We have demonstrated the assembly of 3D structures maintained by continuous energy dissipation through localized optical heating. By quantifying the interplay of different temperature-induced processes - thermophoresis, thermo-osmosis and temperature-based depletion - we were able to explain both the growth dynamics of crystalline structures and the emergence of novel out-of-equilibrium toroidal assemblies. The assembled structures exhibited reconfigurable photonic properties, manifested in tunable stopbands that could be modulated by precise control of the temperature field. Remarkably, these 3D photonic crystals could be assembled within minutes, much faster than conventional approaches.^41–43^ The rapid assembly combined with the ability to dynamically reconfigure the structures through temperature control demonstrates the potential of thermofluidic assembly for the creation of adaptive functional materials. This approach opens up new possibilities for the design of dynamic structures that can respond to external stimuli—a capability that cannot be readily achieved with conventional top-down or equilibrium self-assembly techniques.

## Materials and Methods

### Sample Preparation

The sample consists of a liquid film of 5 μm thickness containing a solution of polystyrene (PS) nanoparticles in 7% (w/v) PEG-6000 (11.67 mM) (along with 5 μm spacers). A mixture of Rhodamine-6G (10^–6^ M) and Nile Blue (10^–5^ M) was further added to the samples where a fluorescent signal was necessary for the measurement of photonic stopbands. The liquid sample lies between two glass coverslips, with the bottom coverslip coated with a 50 nm thin Gold film to facilitate laser-induced heating. Polystyrene particles of 3 different sizes were purchased from Microparticles GmbH and Dynamic Light Scattering (Malvern Zetasizer Ultra) measurements of the particles revealed average diameters of 345, 400 and 442 nm with polydispersities of 0.006, 0.040, and 0.123 respectively. The height of the liquid film maintained by the spacers was several times the diameter of the nanoparticles, providing space for growth in 3 dimensions. Further, the dense colloidal solution used (0.2% (w/v)) facilitated the assembly of a larger and 3-dimensional structure.

### Experimental Setup

The experimental setup consists of an inverted microscope (Olympus, IX71) with an oil-immersion objective lens (Olympus, UPlanApo ×100, NA 0.5–1.35) focusing a CW 532 nm laser beam (CNI, MGL-III-532) onto the sample. The sample is kept on a high precision piezoelectric stage (Physik instrumente, P-733.3 piezo nanopositioner) that allows fine movement in all 3 dimensions. The laser beam passes through an Acousto-Optic Deflector (AA Optoelectronic, DTSXY-400-532), which allows for the accurate positioning and power modulation of the focused laser spot. A LED white light source (Thorlabs, SOLIS-3C) and a Dark-field condenser (Olympus, U-DCW, NA 1.2–1.4) provide dark-field illumination to the sample. After the back-reflected laser-wavelength is rejected from the collected signal using a notch-filter, the light is projected onto the camera (EMCCD, Andor iXon), which images the real plane. The excited fluorescence of the dye mixture is probed in the Fourier plane. The Fourier plane (FP) imaging and spectroscopy is performed by a 4f relay configuration,^28^ which images the Back focal plane of the Objective. The FP image is projected onto an adjustable slit. When the slit is fully open, it allows the camera (sCMOS) to capture the image of the Fourier plane. The FP image contains angle resolved information on the fluorescence signal that passes through the sample. Wavelength dependent FP imaging is performed using band-pass filters centered around 565 nm (565WB20), 590 nm (590BP35) and 660 nm (660BP20) wavelength. To obtain additional spectroscopic information, the light is projected onto the camera through a blazed grating (which is flipped on), while using a narrow slit width.

## Supplementary Material









## Notes

Temperature measurements with 5CB; Numerical temperature profile simulation; numerical simulation of thermo-osmotic flow fields; diffusion coefficient close to surfaces; free convection and Rayleigh number; modeling growth rate of crystal; determination of number density of colloidal particles; numerical simulation of photonic band structures (PDF)
Movie 1: assembly of 345 nm polystyrene particles using a steady laser power of *P*_0_ = 0.26 mW (MP4)
Movie 2: two-stage assembly of 345 nm polystyrene particles using an elevated laser power of *P*_1_ = 2.16 mW for 76 s, followed by a power reduction to *P*_0_ (MP4)
Movie 3: assembly of 345 nm polystyrene particles using pulsed laser heating, alternating between *P*_1_ and *P*_0_ with a 10 s pulse period (MP4)
