# Supplementary material for: Thermofluidic Nonequilibrium Assembly of Reconfigurable Functional Structures
Source: ACS Nano. 2025 Jun 3;19(23):21820–9. doi: 10.1021/acsnano.5c05766 (PMC12177947; doi:10.1021/acsnano.5c05766)
Supplement: Supplementary file 1 [file nn5c05766_si_001.pdf]

Thermofluidic non-equilibrium assembly of reconfigurable functional structures

Desmond Joseph Quinn, Diptabrata Paul, Frank Cichos*
Molecular Nanophotonics Group, Leipzig University, 04103 Leipzig, Germany

CONTENTS

I. Temperature measurements with 5CB 2

II. Numerical temperature profile simulation 2

III. Numerical simulation of thermo-osmotic flowfields 3

IV. Diffusion coefficient close to surfaces 4

V. Free Convection and Rayleigh number 5

VI. Modeling growth rate of crystal 5

VII. Determination of number density of colloidal particles 6

VIII. Numerical simulation of photonic bandstructure 7

References 8

* Frank Cichoscichos@physik.uni-leipzig.de

I. TEMPERATURE MEASUREMENTS WITH 5CB

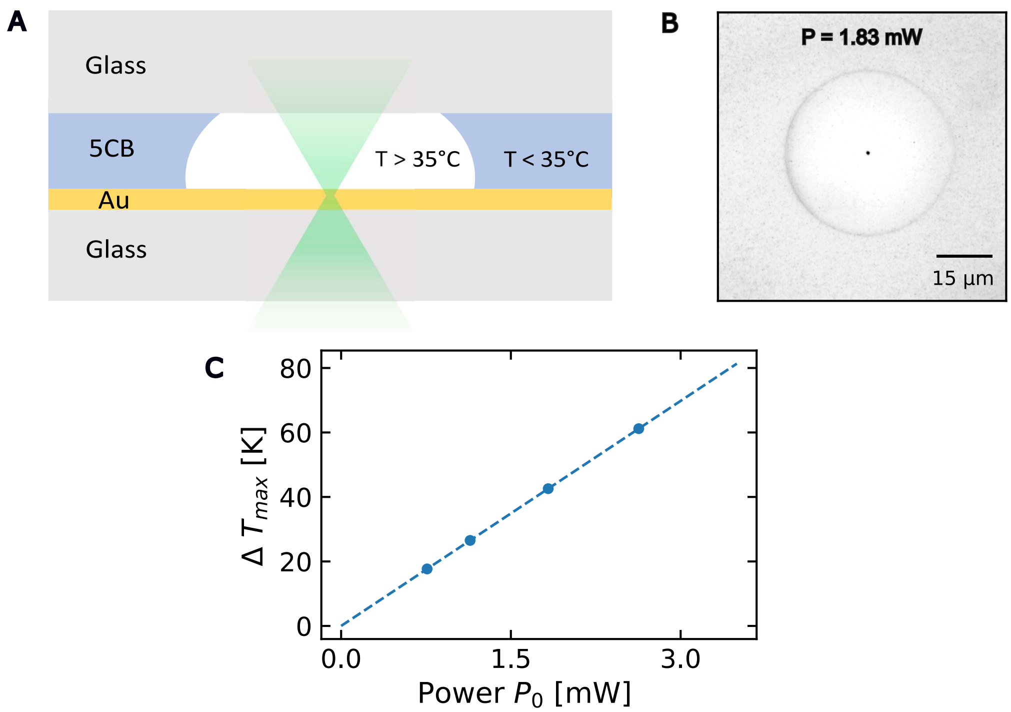

FIG. 1. **Experimental measurement of temperature** (A) The sample geometry of the 5CB liquid film. (B) Image of the 5CB phase boundary at $P = 1.83mW$. (C) The measured temperature increments for different laser powers

The temperature measurement method we use is based on the phase transition of the 5CB (4-Cyano-4'-pentylbiphenyl) liquid crystal. This liquid crystal has a known phase transition temperature of $T_{\rm pt} = 35°C$. Below the transition temperature $T_{\rm pt}$, the liquid crystal exists in the nematic phase where the molecules are stacked and oriented in a preferred direction. Above this temperature, the order is broken and it exists in the isotropic phase. When an object embedded in 5CB is optically heated beyond the phase transition temperature, an isotropic phase forms around the object wherever the temperature exceeds this threshold. The boundary between the isotropic and nematic phases can be clearly observed using dark field microscopy due to the difference in refractive index between the two phases. In the case of radially symmetric temperature profiles, the phase boundary appears in the images as a ring of radius $R_{\rm pt}$.

The temperature increment of 5CB is given by:

$$\Delta T_{\rm max}^{\rm 5CB} = \frac{35° - T_0}{P_0^{\rm pt}} P_0 \tag{1}$$

The temperature increment of water is related to the temperature increment in 5CB as $\Delta T_{\rm max}^{\rm H_2O} = 0.9\,\Delta T_{\rm max}^{\rm 5CB}$. The factor 0.9 accounts for the difference in thermal conductivity of water and 5CB.

From the experiments, it was found that $\Delta T_{\rm max}^{\rm H_2O} = 23.1\,{\rm K}$ at $1\,{\rm mW}$.

II. NUMERICAL TEMPERATURE PROFILE SIMULATION

The temperature distribution due to the laser-heated gold film is obtained by finite-element numerical simulations using the Heat Transfer module in COMSOL Multiphysics. The sample geometry was modeled

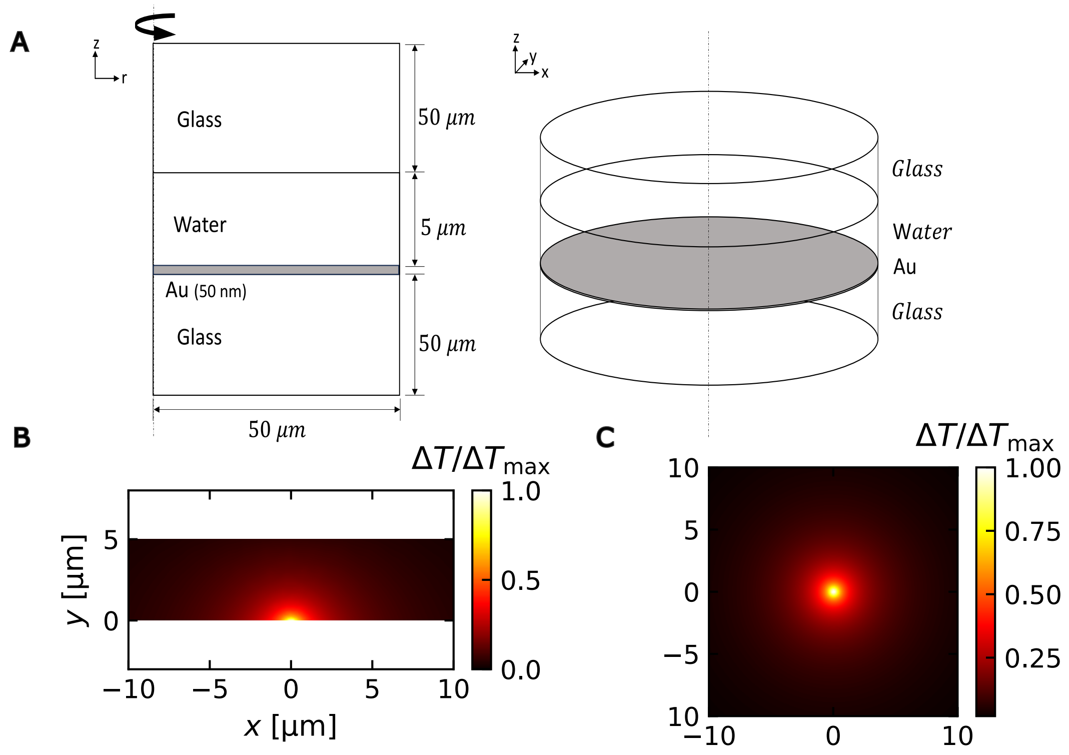

FIG. 2. **Numerical temperature simulation** (A) Sketch of the sample geometry. (B) Normalized temperature profile cross-section. (C) Normalized temperature profiles top-view

by a 2D axisymmetric model. The heating due to the focused laser beam is modeled by defining a heat source within the gold film domain using the equation:

$$Q(r,z) = P_0(1-R)\frac{2\alpha}{\pi w_0^2} e^{\frac{-2r^2}{w_0^2}} e^{-\alpha z} \tag{2}$$

where P_0 is the incident power, λ is the wavelength of the laser (here 532 nm), R and α are the Optical Reflectance and Absorption coefficient of Gold at the laser wavelength (here, 0.6 and $5.3 \cdot 10^7\,\mathrm{m^{-1}}$ respectively), and w_0 is the beam diameter (here, 0.28 µm). The material properties used in the COMSOL simulation are as follows:

Material	κ [$\mathrm{W\,m^{-1}\,K^{-1}}$]	ρ [$\mathrm{g\,cm^{-3}}$]	c_p [$\mathrm{J\,kg^{-1}\,K^{-1}}$]
Glass	1.2	2.2	830
Au	150	19.3	129
Water	0.6	1.0	4182

III. NUMERICAL SIMULATION OF THERMO-OSMOTIC FLOWFIELDS

We combine the heat transfer module with the laminar flow module of COMSOL to simulate the thermo-osmotic flows. The thermo-osmotic flow is modeled as a slip flow by defining the lower boundary as a wall with a slip velocity:

$$v_{\mathrm{TO,slip}} = \frac{\sigma_T \eta}{\rho} \frac{\nabla_{||} T}{T} \tag{3}$$

where σ_T is a dimensionless thermal slip coefficient, η is the viscosity (here $1 \cdot 10^{-3}\,\mathrm{Pa\,s}$), and ρ is the density (here $1000\,\mathrm{kg\,m^{-3}}$) of water. The thermal slip coefficient σ_T is related to the thermo-osmotic coefficient χ

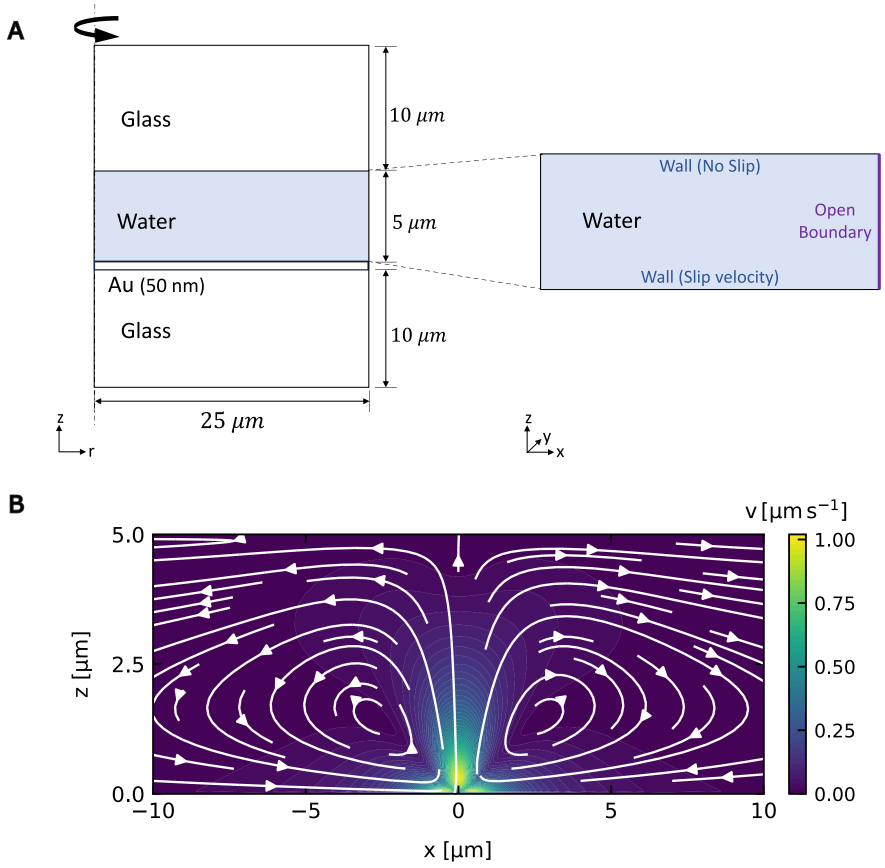

FIG. 3. **Numerical flow field geometry** (A) Sketch of the geometry used for flow field simulations in COMSOL. (B) Numerically simulated Thermo-osmotic flow fields with normalized velocities

as:

$$\sigma_T = \chi \cdot 10^6 \mathrm{m}^{-2}\mathrm{s}$$

In our simulation, we use $\chi = 10 \cdot 10^{-10}\mathrm{m}^2\mathrm{s}^{-1}$ for a gold-water interface.

IV. DIFFUSION COEFFICIENT CLOSE TO SURFACES

The Stokes-Einstein relation defines the diffusion coefficient of particles to be:

$$D_0 = \frac{k_B T}{f_0} \tag{4}$$

where k_B is the Boltzmann constant, T is the temperature, and $f_0 = 6\pi\eta R$ is the friction coefficient for a spherical particle of radius R in bulk solution. However, near the vicinity of a wall, the particle experiences increased friction resulting in lower diffusion coefficients [1].

The correction factor for the in-plane friction coefficient close to a wall $\gamma_{||}$ (where $f_{||} = f_0\gamma_{||}$) is modified as:

$$\gamma_{||}^{-1}(z) = \frac{D_{||}}{D_0} \approx 1 - \frac{9}{16}\frac{R}{z} + \frac{1}{8}\left(\frac{R}{z}\right)^3 \pm \ldots \tag{5}$$

where $D_{||}$ is the colloid's in-plane diffusion coefficient, and z denotes the distance from the particle center to the surface of the wall.

The correction factor for the out-of-plane friction coefficient close to a wall $\gamma_\perp$ (where $f_\perp = f_0\gamma_\perp$) is modified as:

$$\gamma_\perp^{-1}(z) = \frac{D_\perp}{D_0} = \left(\frac{4}{3}\sinh(\alpha)\sum_{n=1}^{\infty}\frac{n(n+1)}{(2n-1)(2n+3)}\left[\frac{2\sinh((2n+1)\alpha)+(2n+1)\sinh(2\alpha)}{4\sinh^2((n+\frac{1}{2})\alpha)-(2n+1)^2\sinh^2(\alpha)}-1\right]\right) \quad (6)$$

where $\alpha = \operatorname{arccos} z/R$

When the particle is confined between two surfaces, the correction factors for the friction coefficients can be approximated as a linear superposition given as:

$$\gamma^{\mathrm{II}}(z) = \gamma^{\mathrm{I}}(z) + \gamma^{\mathrm{I}}(H-z) - 1 \quad (7)$$

where $\gamma^{\mathrm{I}}(z)$ represents either $\gamma_\parallel^{-1}(z)$ or $\gamma_\perp^{-1}(z)$.

V. FREE CONVECTION AND RAYLEIGH NUMBER

Free convection, or natural convection, is a spontaneous flow arising from buoyancy forces within the liquid due to mass density gradients. The most common trigger for these gradients are temperature fields, since the density of a fluid is dependent on the temperature, generally decreasing (due to fluid expansion) with increasing temperatures. The occurrence of the free convective flows depends on the relative magnitude of the buoyancy and viscous forces in the fluid, which is quantified by the dimensionless parameter called the Rayleigh number Ra_L [2].

$$Ra_L = \frac{g\beta\Delta T L^3}{v\alpha} \quad (8)$$

where $g = 9.8\,\mathrm{m\,s^{-2}}$ is the acceleration due to gravity, $\beta = 210\times10^{-6}$ is the coefficient of thermal expansion, ΔT is the temperature increment, $L = 5\,\mathrm{\mu m}$ is the height of the sample chamber, $v = 0.89\,\mathrm{mm^2\,s^{-1}}$ is the kinematic viscosity, and $\alpha = 0.143\,\mathrm{mm^2\,s^{-1}}$ is the thermal diffusivity of water at about 25°C.Inserting the maximum temperature increment in the sample still gives us a Rayleigh number Ra_L on the order of $\sim 10^{-5}$.

The critical Rayleigh number $Ra_{L,c}$, which determines the onset of convective flows has a value of 1708 for our geometry [2], way above the values we could have in our sample. Hence, convective flows are negligible in our sample.

VI. MODELING GROWTH RATE OF CRYSTAL

The colloidal crystal is driven by the particle flux $\mathbf{j}$, which can be written as:

$$\mathbf{j} = c_\mathrm{c}\mathbf{v}_\mathrm{c} \quad (9)$$

where c_c is the number density of particles, and $\mathbf{v}_\mathrm{c}$ is the velocity of the particles.

The number of particles in the crystal at any given time t' is:

$$\mathrm{N}(t') = \frac{\text{Volume of cluster}}{\text{Volume of a single particle}} \times \eta_{\mathrm{pack}} = \frac{(1/2)\times(4/3)\pi r^3(t')}{(4/3)\pi {R_c}^3} \times \eta_{\mathrm{pack}} = \frac{r^3(t')\,\eta_{\mathrm{pack}}}{2\,{R_c}^3} \quad (10)$$

where η_{pack} is the packing fraction of the crystal, $r(t')$ is the radius of the cluster at time t', and R_c is the radius of the colloidal particles being assembled.

Additionally, the total number of particles in the cluster can also be found by integrating the fluxes over the outer surface of the cluster for the time interval of the growth:

$$N(t') = \int_0^t \iint_S \mathbf{j}\cdot\mathbf{dA}\,dt' = \int_0^t \iint_S c_\mathrm{c}\mathbf{v}_\mathrm{c}\cdot\mathbf{dA}\,dt' \quad (11)$$

For a point heat source, the surface of integration represents an isotherm with uniform velocity in the direction perpendicular to the surface, resulting in:

$$N(t') = \int_0^t c_c v_c \iint_S dA\, dt' \tag{12}$$

In our geometry, the area of this surface is given by half the surface area of a sphere having radius $r(t)$. So,

$$N(t') = \int_0^t c_c v_c 2\pi r^2(t')\, dt' \tag{13}$$

Since the velocities are proportional to the temperature gradients, they can be approximated as $v_c = \frac{v_c^0}{r^2}$ for a point heat source whose temperature is known to decay as $\frac{1}{r}$, giving:

$$N(t') = \int_0^t c_c \frac{v_c^0}{r^2(t')} 2\pi r^2(t')\, dt' = \int_0^t c_c v_c^0 2\pi\, dt' = c_c v_c^0 2\pi t \tag{14}$$

Combining Eq. 10 and Eq. 14, one obtains an expression for the radius of the cluster:

$$r(t) = \left(\frac{4\pi {R_c}^3 v_c^0 c_c t}{\eta_{\text{pack}}} \right)^{1/3} \tag{15}$$

and the resulting area would be given by:

$$A(t) = \pi \left(\frac{4\pi {R_c}^3 v_c^0 c_c t}{\eta_{\text{pack}}} \right)^{2/3} = \left(\frac{4\pi^{5/2} {R_c}^3 v_c^0 c_c t}{\eta_{\text{pack}}} \right)^{2/3} \tag{16}$$

The above equation can be written in the more general form:

$$A(t) = (k\, t)^{2/3} \tag{17}$$

indicating that the area of the crystal grows with time following a power law with a growth constant k given by:

$$k = \frac{4\pi^{5/2} {R_c}^3 v_c^0 c_c}{\eta_{\text{pack}}} \tag{18}$$

VII. DETERMINATION OF NUMBER DENSITY OF COLLOIDAL PARTICLES

The number density of the colloidal particles c_c is given by:

$$c_c = \frac{\mathrm{N}}{\mathrm{FoV} \times \mathrm{DoF}} \tag{19}$$

where N corresponds to the number of particles detected in the first frame, FoV is the Field of View corresponding to the spatial extent of the imaged plane, and DoF is the Depth of Field corresponding to the depth of the sample space that appears focused in the image and can be approximated as $\mathrm{DoF} = \frac{\lambda\, n}{NA^2}$, where $\lambda = 0.590\,\mu\mathrm{m}$ is taken as the average wavelength of the illumination used, $n = 1.33$ is the refractive index of the medium, and $NA = 1.2$ is the numerical aperture used [3]. The number of particles N was detected using the Difference of Gaussian method [4][5] and was found to be 496, resulting in a number density of $0.76\,\mu\mathrm{m}^{-3}$.

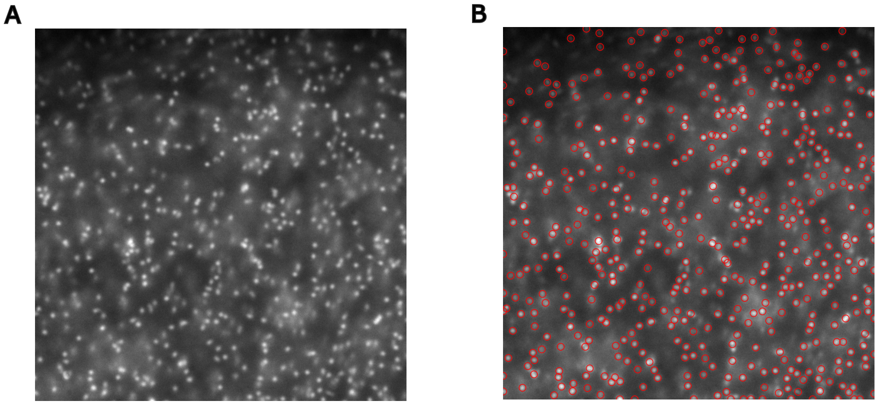

FIG. 4. **Number density determination** (A) Microscope image of sample of colloidal particles. (B) Overlay of the particle outlines detected with the Difference of Gaussians method.

VIII. NUMERICAL SIMULATION OF PHOTONIC BANDSTRUCTURE

The propagation of light inside the 3D crystal structure made up of Polystyrene particles in water could be described by the following simplified form of Maxwell's equations for time harmonic electromagnetic fields:

$$\nabla \times \left(\frac{1}{\epsilon(r)} \nabla \times \mathrm{H(r)} \right) = \left(\frac{\omega}{c} \right)^2 \mathrm{H(r)}$$

Solving the above eigenvalue equation for such a periodic structure, one obtains certain modes that are forbidden. The set of frequencies (modes) not allowed to propagate in the photonic crystal is referred to as the band gap, and the periodic structure is called a photonic crystal. In cases where the prohibited frequencies are angle dependent, we use the term stopband. To visualize these bands, we obtained the photonic band structure of our assembled crystal by numerically solving the above equation with the MPB package [6]. We compute the bands for wavevectors corresponding to the high symmetry points, i.e., the corners in the irreducible Brillouin zone of the FCC lattice. The lattice geometries are specified in dimensionless units.

Symmetry points (u, v, w)	$[k_x, k_y, k_z]$
$\Gamma : (0,0,0)$	$[0,0,0]$
$X : (0, \frac{1}{2}, \frac{1}{2})$	$[0, 2\pi/a, 0]$
$L : (\frac{1}{2}, \frac{1}{2}, \frac{1}{2})$	$[\pi/a, \pi/a, \pi/a]$
$W : (\frac{1}{4}, \frac{3}{4}, \frac{1}{2})$	$[\pi/a, 2\pi/a, 0]$
$U : (\frac{1}{4}, \frac{5}{8}, \frac{5}{8})$	$[\pi/2a, 2\pi/a, \pi/2a]$
$K : (\frac{3}{8}, \frac{3}{4}, \frac{3}{8})$	$[3\pi/2a, 3\pi/2a, 0]$

We observed a stopband in the ΓL direction, which corresponds to propagation of light in the (111) plane under certain specific angles of incidence (wavevectors).

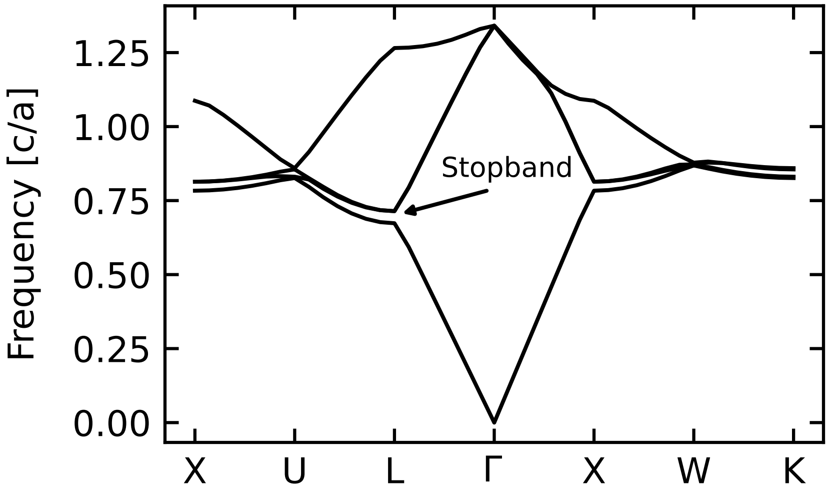

FIG. 5. **Photonic stopband simulation** The simulated stopband for an FCC crystal obtained from MPB simulations.

[1] J. Happel and H. Brenner, “Low reynolds number hydrodynamics (mechanics of fluids and transport processes),” *Martinus Nijhoff*, 1973.

[2] F. P. Incropera, D. P. DeWitt, T. L. Bergman, A. S. Lavine, *et al.*, *Fundamentals of heat and mass transfer*, vol. 6. Wiley New York, 1996.

[3] M. Bass, E. Van Stryland, D. Williams, and W. Wolfe, *Handbook of optics, Volume II: Devices, Measurements, Properties, sources and detectors, radiometry and photometry.* McGraw-Hill, Inc., 1994.

[4] J. C. Crocker and D. G. Grier, “Methods of digital video microscopy for colloidal studies,” *Journal of Colloid and Interface Science*, vol. 179, no. 1, pp. 298–310, 1996.

[5] N. C. K. v. d. W. M. C. D. B. Allan, T. Caswell and R. Verweij, “trackpy.” “soft-matter/trackpy: Trackpy v0.5.0”. Zenodo, Apr. 13, 2021.

[6] S. G. Johnson and J. D. Joannopoulos, “Block-iterative frequency-domain methods for maxwell’s equations in a planewave basis,” *Optics express*, vol. 8, no. 3, pp. 173–190, 2001.
